# Rigidifying Flexible Regions of a Bacterial Laccase Enables High-Temperature Aflatoxin B1 Degradation

**DOI:** 10.3390/microorganisms14040856

**Published:** 2026-04-10

**Authors:** Dongwei Xiong, Huiying Sun, Yuhang Sun, Peng Li, Miao Long

**Affiliations:** Key Laboratory of Livestock Infectious Diseases, Ministry of Education, College of Animal Science & Veterinary Medicine, Shenyang Agricultural University, Shenyang 110866, China; 2022200183@stu.syau.edu.cn (D.X.); 2022240765@stu.syau.edu.cn (H.S.); syh2019@syau.edu.cn (Y.S.); lipeng2018@syau.edu.cn (P.L.)

**Keywords:** Aflatoxin B1, laccase, thermostability, semi-rational design, molecular dynamics simulation

## Abstract

Aflatoxin B1 (AFB1) poses a serious threat to global food and feed safety. Laccase-based enzymatic degradation represents a promising green strategy for AFB1 removal; however, its industrial application is severely limited by the rapid thermal inactivation of wild-type enzymes under high-temperature processing conditions (>70 °C). Here, we engineered the thermal stability of a laccase from *Bacillus amyloliquefaciens* B10 through an integrated strategy combining computational structural biology with semi-rational design. By coupling molecular dynamics (MD) simulations with folding free-energy (ΔΔG) calculations, we identified key flexible regions associated with thermal instability and subsequently implemented iterative saturation mutagenesis. The best single mutant, R196C, retained more than 96% relative activity after heat treatment at 80 °C for 10 min. Further iterative mutational stacking progressively enhanced thermostability: the R90E/R196C double mutant showed 1.25-fold higher activity at 80 °C than R196C, and the R90E/R196C/H54F triple mutant showed a further 1.16-fold increase over the double mutant. The final quadruple mutant, R90E/R196C/H54F/R253I, achieved 86.9% AFB1 degradation at 80 °C after 24 h. High-temperature MD simulations (100 ns at 353.15 K) indicated that the enhanced thermostability was associated with reduced conformational flexibility, lower radius of gyration (Rg) and solvent-accessible surface area (SASA), and a coil-to-β-sheet transition that contributed to stabilization of the protein core. In addition, efficient secretory expression of the engineered enzyme was achieved in *Pichia pastoris*, reaching 3.0 U/mL, while the crude enzyme maintained more than 70% activity at 80 °C. Collectively, these results provide a practical basis for the rational engineering and scalable production of thermostable biocatalysts for AFB1 detoxification-related applications of AFB1 control, and offer broader insights into the targeted enhancement of thermal stability in industrial enzymes.

## 1. Introduction

Under the combined influence of increasingly integrated global agricultural trade networks and worsening climate change, the distribution of mycotoxin contamination has exhibited a pronounced pattern of geographic expansion [1,2]. Among the several hundred mycotoxins that have been isolated and characterized, AFB1 has received particular attention owing to its potent carcinogenic and cytotoxic effects [3]. Molecular toxicology studies have shown that, after entering the circulation, AFB1 undergoes extensive oxidative biotransformation in the liver via cytochrome P450 enzymes, including CYP3A4 and CYP1A2, to form the highly electrophilic AFB1-8,9-epoxide, which irreversibly binds to guanine residues at the N7 position of nuclear DNA, ultimately leading to tumour suppressor gene inactivation and hepatocellular carcinoma [4]. Conventional physical and chemical detoxification strategies suffer from substantial drawbacks because the distinctive dihydrofuran and coumarin lactone ring structures of AFB1 endow it with remarkable physicochemical resistance, including a melting point as high as 200–300 °C; as a result, standard thermal extrusion treatments (e.g., 150 °C) are often insufficient for complete detoxification and may degrade thermolabile feed nutrients, while chemical treatments, such as ammoniation and ozonation, carry risks of residual chemicals and equipment corrosion [5].

Accordingly, biocatalytic AFB1 transformation strategies characterized by mild reaction conditions, strong substrate specificity, and minimal disruption of the native nutritional composition of the treated matrix have emerged as a major focus of both academic and industrial research in recent years. To date, multiple microbial enzymes have been shown to degrade AFB1, including F420-dependent reductases, manganese peroxidases, lactonases and laccases [6,7,8]. Among these, laccase, a multicopper oxidase, catalyses substrate oxidation by directly using molecular oxygen as the sole electron acceptor and producing water as the only by-product, without requiring expensive and hazardous cofactors such as hydrogen peroxide, thus highlighting its considerable commercial promise [9]. Mass spectrometric and metabolomic analyses indicate that laccase detoxifies AFB1 primarily by targeting its two key toxicophoric moieties—promoting double-bond cleavage within the dihydrofuran ring or triggering lactone ring opening—thereby converting it into low-toxicity or non-toxic derivatives, such as AFQ1 and AFD1 [10]. In contrast to fungal laccases, which are generally limited to mildly acidic and ambient-temperature conditions, bacterial laccases derived from *Bacillus* spp., exemplified by CotA, inherently exhibit broader pH tolerance and superior baseline thermostability, making them the preferred chassis for AFB1 transformation enzyme engineering [11].

Although native laccases show excellent AFB1-degrading activity in laboratory-scale aqueous systems at ambient temperature, they face a profound thermodynamic barrier in actual industrial applications. In modern large-scale feed and food production, raw materials are routinely subjected to pelleting, extrusion and high-temperature sterilization, with transient process temperatures frequently reaching 70–90 °C or above. Under such severe thermal stress, the non-covalent interaction network responsible for maintaining the three-dimensional structure of most wild-type laccases rapidly disintegrates, thereby triggering unfolding, denaturation and aggregation-mediated loss of activity [12,13]. Although conventional encapsulation technologies used in industry can confer physical thermal protection, they frequently delay enzyme release in the gastrointestinal tract of animals, thereby missing the optimal timing for enzymatic action and markedly diminishing biological efficacy [14]. Therefore, re-engineering the laccase molecule at the genetic level to improve its intrinsic resistance to elevated temperatures represents an important prerequisite for future application in thermally demanding feed-processing systems, although further process-level validation under realistic industrial conditions is still required. In recent years, computer-aided semi-rational design has become a new paradigm in enzyme engineering. This approach integrates all-atom molecular dynamics simulations to accurately identify thermally sensitive flexible regions within the protein, and subsequently combines folding free-energy calculations with virtual saturation mutagenesis to greatly enhance engineering efficiency [15,16,17]. A survey of recent progress in the engineering of mycotoxin-degrading laccases indicates that, despite notable breakthroughs, most studies have concentrated on local optimization of regions surrounding the substrate-binding pocket to enhance catalytic efficiency. Liu et al. showed in 2024 that the Q441A variant of CotA laccase from *Bacillus licheniformis* enhanced catalytic efficiency toward AFB1 by 1.73-fold through reducing local steric hindrance [18]. Moreover, saturation mutagenesis at position T418 in the loop region adjacent to the T1 copper centre of the substrate-binding cavity in the frL103 laccase from *Bacillus vallismortis*, exemplified by T418A and T418S, successfully remodelled the local hydrogen-bond network and enhanced substrate affinity [19]. However, these frontier studies have largely been limited to fine adjustment of the catalytic centre, and there remains a substantial knowledge gap in the systematic evolution and mechanistic dissection of laccases based on global conformational dynamics, particularly through ISM (Iterative Saturation Mutagenesis)-guided engineering to withstand extreme thermal conditions above 80 °C while maintaining prolonged high-efficiency detoxification.

Building on the scientific context described above, this study chose a wild-type laccase from *Bacillus amyloliquefaciens* with intrinsic AFB1-degrading activity as the starting template for extensive computationally guided molecular engineering. By integrating all-atom molecular dynamics (MD) simulations with conformational energy minimization, we precisely identified flexible sites in B10 laccase and applied an iterative ISM strategy to enable synergistic multi-site recombination. The resulting mutants were then systematically characterized for their kinetic behaviour at elevated temperatures and their capacity to degrade AFB1, and a high-density eukaryotic secretory expression platform based on *Pichia pastoris* was subsequently developed for efficient production of the engineered laccase.

## 2. Materials and Methods

### 2.1. Strains, Plasmids, and Reagents

The wild-type *Bacillus amyloliquefaciens* B10 strain used in this study was isolated and maintained in our laboratory. Competent cells of the cloning host *Escherichia coli* DH5α and the prokaryotic expression host *Escherichia coli* BL21(DE3), together with Ni Sepharose 6 Fast Flow affinity chromatography medium, were obtained from Sangon Biotech (Shanghai, China). Competent *Pichia pastoris* GS115 cells were obtained from Wuhan Piont Biotechnology Co., Ltd. (Wuhan, China). The prokaryotic expression vector pET-28a and the eukaryotic expression vector pPICZalphaA were routinely preserved in our laboratory. The AFB1 standard was obtained from Sigma (St. Louis, MO, USA), whereas other molecular biology reagents, including T4 DNA ligase, the restriction enzymes PmeI, DpnI, BamHI, XhoI, EcoRI and XbaI, as well as the site-directed mutagenesis kit, were purchased from TaKaRa (San Jose, CA, USA) or Vazyme Biotech Co., Ltd. (Nanjing, China).

### 2.2. Structural Modeling and All-Atom Molecular Dynamics Simulation

The three-dimensional structure of wild-type B10 laccase (ASB53002.1) was constructed by homology modelling. A homologous template was identified by searching the Protein Data Bank using NCBI BLAST (https://blast.ncbi.nlm.nih.gov/Blast.cgi, accessed on 13 May 2024), and the crystal structure deposited under PDB ID 6T0Y, sharing 49.40% sequence identity with the target protein, was selected as the modelling template. Structural alignment between the target model and the template was performed in PyMOL v2.5.4 (Schrödinger, Inc., New York, NY, USA), the template Zn^2+^ ion was retained, and residues around the predicted Zn^2+^-binding site were manually adjusted accordingly. The recombinant laccase structure was then prepared using MGLTools 1.5.7 (Scripps Research Institute, San Diego, CA, USA) by adding hydrogen atoms and atomic charges to generate the corresponding pdbqt file. Molecular dynamics simulations were carried out in GROMACS 2020.6 (KTH Royal Institute of Technology and Stockholm University, Stockholm, Sweden) using the CHARMM36m (University of Maryland, Baltimore, MD, USA) force field. The protein was placed in a cubic box containing TIP3P water molecules, with a minimum distance of 1.0 nm between the protein and the box edge. Periodic boundary conditions were applied in all three dimensions. The system was neutralized by adding counterions, and NaCl was further added to a final concentration of 0.15 M. Energy minimization was performed using the steepest descent algorithm to eliminate steric clashes and atomic overlaps. The system was subsequently equilibrated under NVT and NPT ensembles. Production simulations were then performed for 100 ns at 353.15 K (80 °C) and 1 bar under isothermal–isobaric conditions, using a 2 fs integration time step. After the simulations, trajectory data were extracted to analyse the root mean square deviation (RMSD), root mean square fluctuation (RMSF), Rg, and SASA. Protein conformational changes were visualized, and secondary-structure analysis was performed, using PyMOL v2.5.4 and PDBsum (EMBL-EBI, Hinxton, UK; https://www.ebi.ac.uk/thornton-srv/databases/pdbsum/, accessed on 14 May 2024).

### 2.3. Computer-Aided Semi-Rational Design and Folding Free-Energy Calculations

Based on the RMSF profiles obtained from the molecular dynamics simulations described in Section 2.2, the intrinsically disordered N-terminal residues Met1 and Asn2 were excluded from further analysis. Residues showing high internal flexibility and large-amplitude fluctuations, including Lys217, Glu216, Arg196, Arg90, Arg253, Arg56 and Gly218, were selected as initial screening targets. Virtual saturation mutagenesis was subsequently performed for these residues using the Calculate Mutation Energy (Stability) module in Discovery Studio 2019 (BIOVIA, San Diego, CA, USA) under acidic conditions (pH 3). The mutation-induced change in folding free energy (ΔΔG) was calculated for each substitution, and variants with negative predicted mutation energies were considered potentially beneficial for thermostability and selected for subsequent experimental validation.

### 2.4. Construction of the Recombinant Plasmid pET-28a-B10

Strain B10 was grown overnight in LB medium (Sangon, Shanghai, China), and genomic DNA was extracted using a DNA extraction kit (TaKaRa, Tokyo, Japan) for subsequent experiments. BamHI and XhoI (TaKaRa, Tokyo, Japan) were used as the restriction enzymes, and pET-28a served as the expression vector; the primers used for amplification of the target fragment were as follows: Lac-F: 5′-CGGGATCCATGAATACATATCACCCGTTTAGTCTT-3′, Lac-R: 5′-CCCTCGAGTTATGCCTCCTTCATTCCGATAAAG-3′. PCR was carried out under the following conditions: 98 °C for 10 s, 55 °C for 5 s, and 72 °C for 30 s, for a total of 34 cycles, followed by holding at 4 °C. The amplified PCR fragment was purified using an agarose gel DNA extraction kit (Thermo Fisher Scientific, Waltham, MA, USA). The B10 laccase gene fragment digested with both restriction enzymes was inserted into pET-28a, and the resulting construct was transformed into Trans5α chemically competent cells (TransGen, Beijing, China); transformants were then plated on LB agar containing 100 μg/mL kanamycin and incubated overnight. Three positive clones were selected for sequencing, and the plasmid from the sequence-verified clone showing complete identity to the B10 laccase gene was then isolated and transformed into *E. coli* BL21(DE3) chemically competent cells (TransGen, Beijing, China).

### 2.5. Construction of Iterative Saturation Mutagenesis Libraries and Mutant Cloning

Based on the virtual screening results, mutagenic primers were designed using CE Design software (Vazyme, Nanjing, China; https://tool.vazyme.com:18002/cetool/singlepoint.html, accessed on 25 August 2024), and whole-plasmid amplification was performed on the recombinant plasmid pET-28a-B10 using inverse PCR-based site-directed mutagenesis. The amplification products were treated with DpnI (37 °C for 2 h) to remove the methylated wild-type template, followed by Exnase II (Vazyme, Nanjing, China)-mediated recombinational circularization (37 °C for 30 min), and then transformed into competent *E. coli* DH5α cells. Recombinant clones were screened by PCR and confirmed by plasmid sequencing for successful mutagenesis. After the optimal single-site mutant was identified, it was used as the new template for iterative saturation mutagenesis, through which multiple rounds of pairwise recombination and sequential accumulation of mutations at different sites were carried out, ultimately yielding a panel of combinatorial mutants, including the optimal quadruple mutant R90E/R196C/H54F/R253I.

### 2.6. Prokaryotic Expression and Affinity Purification of the Recombinant Laccase

The recombinant expression plasmid, verified by sequencing, was transformed into competent *E. coli* BL21(DE3) cells. A single colony was inoculated into LB medium supplemented with 50 μg/mL kanamycin and incubated at 37 °C and 170 rpm until the culture reached mid-log phase (OD600 ~0.6). Expression was then induced with IPTG (Solarbio, Beijing, China) at a final concentration of 0.5 mM at 16 °C for 16 h. Cells were collected by centrifugation (8000 rpm for 15 min), resuspended in sterile PBS containing 1 mM PMSF (Solarbio, Beijing, China), and lysed by ultrasonication in an ice bath. After centrifugation, the crude enzyme extract was collected, and the recombinant laccase carrying a 6 × His tag was purified using a pre-equilibrated Ni Sepharose 6 Fast Flow affinity column with stepwise elution using buffers containing 50, 100, 150, 200 and 500 mM imidazole. Fractions containing the target protein were collected, and their purity was assessed by 12.5% SDS–PAGE.

### 2.7. Assessment of Laccase Thermostability and In Vitro AFB1 Degradation

Laccase activity was measured using ABTS [2,2′-azino-bis(3-ethylbenzothiazoline-6-sulfonic acid)] as the substrate and a commercial laccase activity assay kit (Solarbio, Beijing, China), with absorbance monitored at 420 nm. One unit (U) of laccase activity was defined as the amount of enzyme required to oxidize 1 nmol of ABTS per min under the assay conditions, and enzyme activity was expressed as U/mg protein. For thermostability analysis, purified laccase was incubated for 10 min in water baths preset at 40, 50, 60, 70, 80, and 90 °C, rapidly cooled, and the residual activity was subsequently measured; the highest activity obtained under the optimal condition was defined as 100%, and the remaining values were expressed as relative activity. In the in vitro AFB1 degradation assay, the concentration of the purified enzyme was first quantified using a BCA (Solarbio, Beijing, China) assay kit. Purified laccase (final concentration, 20 µg/mL) was incubated in the dark with AFB1 standard solution (final concentration, 5 µg/mL) for 24 h at 40, 50, 60, 70, 80, and 90 °C. All laccase-catalyzed AFB1 conversion assays were carried out in centrifuge tubes. No active aeration or oxygen bubbling was applied during incubation. Therefore, oxygen available for the reaction was provided by the dissolved oxygen initially present in the reaction medium, together with passive gas–liquid exchange through the tube headspace. After incubation, an equal volume of HPLC-grade methanol (Fisher, Waltham, MA, USA) was added to terminate the reaction, and the mixture was vortexed thoroughly and filtered through a 0.22 µm organic-solvent-resistant membrane filter. The concentration of AFB1 in the filtrate was determined by HPLC (Thermo U3000, Thermo Fisher Scientific, Waltham, MA, USA) with fluorescence detection following post-column photochemical derivatization (Pribolab MDS 3000S, Pribolab Pte. Ltd., Qingdao, China). Chromatographic separation was achieved on a C18 column (250 × 4.6 mm, 5 μm, Agilent ZORBAX Eclipse Plus, Agilent Technologies, Santa Clara, CA, USA) using methanol/water (50:50, *v*/*v*) as the mobile phase at a flow rate of 1.0 mL/min. The injection volume was 20 μL, the column temperature was maintained at 30 °C, and fluorescence detection was performed at 365 nm. For quantitative analysis of AFB1 in feed samples, an external standard calibration curve was established using AFB1 standard solutions at concentrations of 1, 2, 5, 10, 20, 50, 100, and 200 μg/kg, which were analyzed under the same HPLC conditions as the samples. The calibration curve was generated by plotting peak area against AFB1 concentration. Under these analytical conditions, the limit of detection (LOD) and limit of quantification (LOQ) for AFB1 were 0.03 μg/kg and 0.1 μg/kg, respectively. The degradation rate was calculated by comparing the characteristic AFB1 peak area of each sample with that of the corresponding control.

### 2.8. Construction and Optimization of a High-Density Secretory Expression System for Pichia Pastoris

The gene sequence encoding the optimal mutant was codon-optimized for *Pichia pastoris*, and EcoRI and XbaI restriction sites were incorporated into the upstream and downstream primers. The target fragment and the pPICZαA vector (Invitrogen, Waltham, MA, USA) were digested with both restriction enzymes and ligated with T4 DNA ligase (Vazyme, Nanjing, China); the resulting recombinant plasmid was linearized with PmeI and introduced into competent *Pichia pastoris* GS115 cells by electroporation (1600 V, 25 µF, 200 Ω, 4 ms). Transformants were screened on MD agar plates (Sangon, Shanghai, China) and verified by PCR using the universal 5′AOX/3′AOX primers. Positive transformants were inoculated into BMGY (Sangon, Shanghai, China) medium and grown at 28 °C and 220 rpm until the culture reached an OD600 of 3.0. Cells were harvested by centrifugation, resuspended in BMMY medium (Sangon, Shanghai, China) for induction, and supplemented with methanol every 24 h. To optimize the expression conditions, a two-factor crossed-gradient experiment was performed using a temperature gradient (25, 28, 30, and 32 °C) and a final methanol concentration gradient (0.5%, 1.0%, 1.5%, and 2.0%). During induction, culture supernatants were collected every 12 h, concentrated and desalted using 35 kDa ultrafiltration devices, and subsequently analyzed for the activity and thermostability of the secreted laccases, as well as their AFB1 degradation performance at different temperatures (40, 50, 60, 70, 80, and 90 °C).

### 2.9. Degradation of AFB1 in Feed Using Culture Supernatant from Pichia pastoris Expressing pPICZαA-R90E/R196C/H54F/R253I

A 2.0 g sample of naturally AFB1-contaminated broiler feed (corn–soybean meal), with an initial AFB1 concentration of approximately 50 μg/kg, was mixed with 10 mL of fermentation supernatant from *Pichia pastoris* expressing pPICZαA-R90E/R196C/H54F/R253I, diluted with PBS to 2.0 U/mL. The control group was treated in parallel with culture supernatant from *P. pastoris* harbouring the empty vector (pPICZαA-Vector), using the same treatment ratio. All reaction mixtures were incubated in the dark at 40 °C and 50 rpm. for 24 h. No active aeration or oxygen bubbling was applied during incubation. Thus, oxygen available for laccase catalysis was provided by the dissolved oxygen initially present in the liquid phase, together with passive gas–liquid exchange through the reaction headspace. Following incubation, feed samples were cleaned up using an AFB1 immunoaffinity column (NCS testing technology, Beijing, China) before chromatographic analysis. The recovery of AFB1 from feed samples was 96.5%.

### 2.10. Statistical Analysis

All quantitative experiments—including enzyme activity assays under different temperature conditions, HPLC-based quantification of in vitro AFB1 degradation, and optimization of eukaryotic fermentation yields—were performed with at least three independent biological replicates (*n* = 3). Data are presented as mean ± standard deviation (mean ± SD), and normality tests were conducted before statistical analysis. Statistical analyses were conducted using SPSS 23.0 software (IBM Corp., Armonk, NY, USA).

## 3. Results

### 3.1. Identification of Conformational Flexibility Hotspots and in Silico Mutation Screening

**Figure 1:**
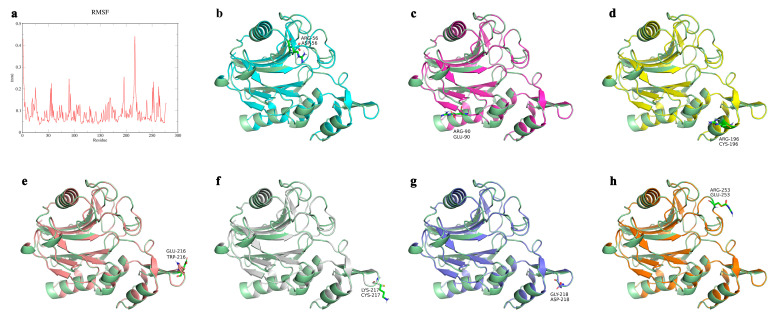
Computational identification of conformational flexibility hotspots and virtual saturation mutagenesis screening in *Bacillus amyloliquefaciens* B10 laccase. (**a**) RMSF profile of B10 laccase obtained from all-atom molecular dynamics simulations in aqueous solution. (**b**–**h**) Cartoon representation of the homology model of wild-type B10 laccase showing the locations of the candidate flexible residues: (**b**) R56D, (**c**) R90E, (**d**) R196C, (**e**) E216W, (**f**) K217C, (**g**) G218D and (**h**) R253E.

The three-dimensional structure of wild-type B10 laccase was constructed by homology modelling using the crystal structure of purine nucleoside phosphorylase (PDB ID: 6T0Y), which shares 49.40% sequence identity, as the template. Preliminary all-atom molecular dynamics simulations were conducted in aqueous solvent using GROMACS, and RMSF profiles were extracted from the simulation trajectories. After excluding the intrinsically flexible disordered N-terminal residues (MET-1 and ASN-2), seven prominent flexibility peaks were identified within B10 laccase based on RMSF analysis (Figure 1): LYS-217 (0.4412 nm), GLU-216 (0.3243 nm), ARG-196 (0.2545 nm), ARG-90 (0.2456 nm), ARG-253 (0.2322 nm), ARG-56 (0.2252 nm) and GLY-218 (0.2246 nm). The candidate residues were further evaluated using the Calculate Mutation Energy (Stability) module in Discovery Studio to calculate the mutation-induced change in folding free energy (ΔΔG). The predicted favourable substitutions and their corresponding ΔΔG values were R56D (−1.64 kcal/mol), R90E (−1.33 kcal/mol), R196C (−2.32 kcal/mol), E216W (−1.02 kcal/mol), K217C (−1.57 kcal/mol), G218D (−0.53 kcal/mol) and R253E (−0.82 kcal/mol) (Figure 1b–h). All candidate mutations displayed negative ΔΔG values, indicating enhanced predicted structural stability.

### 3.2. Catalytic Performance and Thermostability of Single-Site Laccase Variants

**Figure 2:**
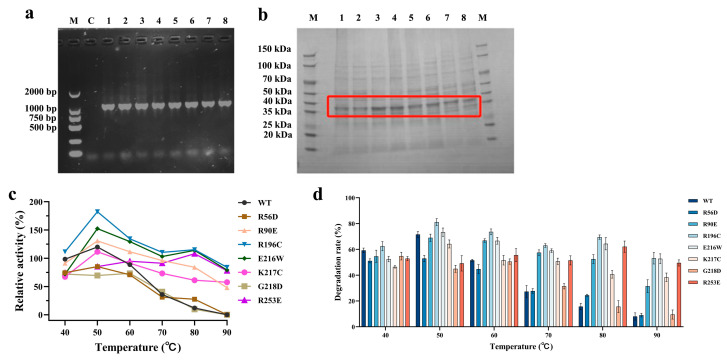
Analysis of the Thermostability and AFB1 Degradation Performance of B10 Laccase Single-Point Mutant. (**a**) Verification of positive clones for the pET-28a-recombinant B10 laccase single-point mutant (M: DNA marker; C: negative control; 1–8: WT, R56D, R90E, R196C, E216W, K217C, G218D, and R253E). (**b**) SDS-PAGE analysis of the B10 laccase single-point mutant (M: protein marker; 1–8: B10-lac, R56D, R90E, R196C, E216W, K217C, G218D, and R253E; The red box highlights the protein band at approximately 35–40 kDa corresponding to the target protein). (**c**) Thermostability of the B10 laccase single-point mutant. (**d**) Effect of temperature on the degradation of AFB1 by B10 laccase single-point mutant. laccase (20 μg/mL), AFB1 (5 μg/mL). The activity of the WT enzyme at 40 °C was set to 100%, and the relative activities of the other samples were normalized accordingly.

Guided by the virtual screening results, seven single-point mutants were generated by inverse PCR-mediated site-directed mutagenesis and successfully expressed and purified in *E. coli* BL21(DE3), with SDS–PAGE showing a single band at approximately 35 kDa (Figure 2a,b). Enzyme activity measurements across 40–90 °C showed that wild-type B10 laccase began to lose activity substantially at 60 °C, retained only minimal residual activity at 80 °C, and was completely inactivated at 90 °C (Figure 2c). By contrast, all seven single-site variants showed enhanced thermostability at elevated temperatures. Among these variants, the R196C mutant showed the best overall performance, displaying the highest absolute activity between 50 and 60 °C and retaining more than 96% of its relative activity after a 10 min heat treatment at 80 °C. In substrate degradation assays, in which mutant laccase (20 μg/mL) and AFB1 (5 μg/mL) were incubated together for 24 h, HPLC analysis showed that the R196C variant achieved an AFB1 degradation rate of 69.5% at 80 °C, significantly exceeding that of the wild type (Figure 2d). The R90E, E216W and R253E variants likewise retained relatively strong degradation activity under high-temperature conditions.

### 3.3. Iterative Saturation Mutagenesis Identifies Multi-Mutant Thermostable Variants

**Figure 3:**
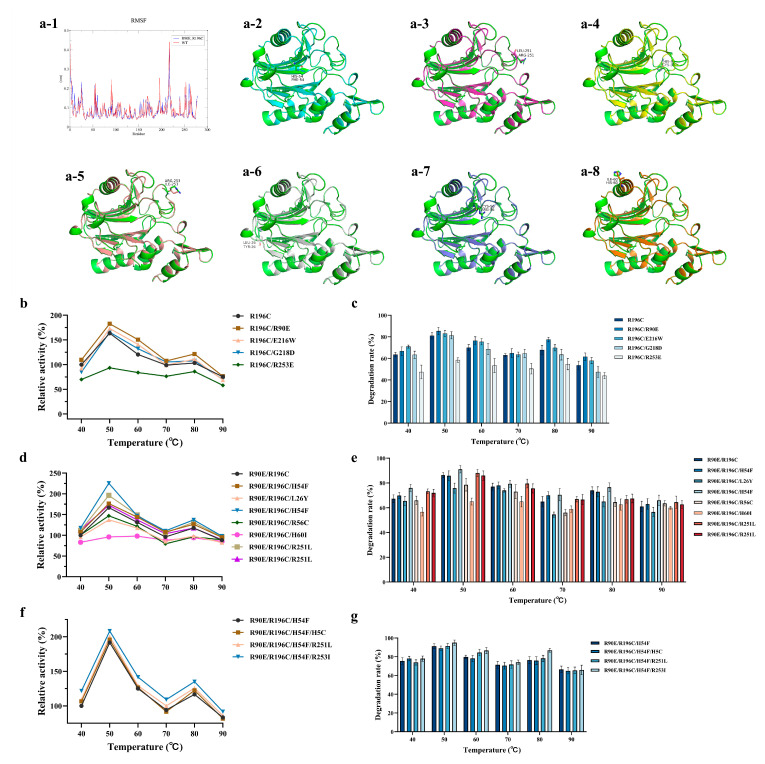
Analysis of the Thermostability and AFB1 Degradation Performance of B10 Laccase Multi-point Mutants. (**a**) Computational identification of conformational flexibility hotspots and virtual saturation mutagenesis screening in the R90E/R196C double mutant. (**a-1**) RMSF profiles of the wild-type enzyme and the R90E/R196C double mutant obtained from molecular dynamics simulations. (**a-2**)–(**a-8**) Structural localization of the candidate residual flexible residues in the R90E/R196C mutant model: (**a-2**) H54F, (**a-3**) R251L, (**a-4**) H5C, (**a-5**) R253I, (**a-6**) L26Y, (**a-7**) R56C and (**a-8**) H60I. (**b**) Thermostability of B10 laccase double-point mutant. (**c**) Effect of temperature on AFB1 degradation by B10 laccase double-point mutant. (**d**) Thermostability of B10 laccase triple-point mutant. (**e**) Effect of temperature on AFB1 degradation by B10 laccase triple-point mutant. (**f**) Thermostability of B10 laccase quadruple-point mutant. (**g**) Effect of temperature on AFB1 degradation by B10 laccase quadruple-point mutant. laccase (20 μg/mL), AFB1 (5 μg/mL). The activity of the R196C enzyme (**b**), R90E/R196C enzyme (**d**), and R90E/R196C/H54F enzyme (**f**) at 40 °C was set to 100%, and the relative activities of the other samples were normalized accordingly.

Using R196C as the starting template, pairwise combinations with R90E, E216W, LYS218 and R253E were constructed to generate a double-mutant library. Activity screening showed that the R90E/R196C double mutant exhibited the strongest synergistic effect, with a specific activity at 80 °C that was 1.25-fold higher than that of the R196C single mutant, and an AFB1 degradation rate of 77.5% under high-temperature conditions (Figure 3b,c). A further round of MD simulation and in silico mutational energy analysis was carried out on the optimal R90E/R196C double-mutant model, identifying seven secondary target substitutions: H54F, R251L, H5C, R253I, L26Y, R56C and H60I (Figure 3a). Following construction and screening of the corresponding triple mutants, R90E/R196C/H54F was identified as the best-performing combination, with its relative activity at 80 °C reaching 1.16-fold that of the double mutant (Figure 3d,e). Finally, further stacking of candidate mutations on this background yielded the quadruple mutant R90E/R196C/H54F/R253I (Figure 3f,g). Activity and degradation assays showed that the quadruple mutant achieved an AFB1 degradation rate of 86.9% at 80 °C.

### 3.4. Structural Evolution During Molecular Dynamics Simulations

**Figure 4:**
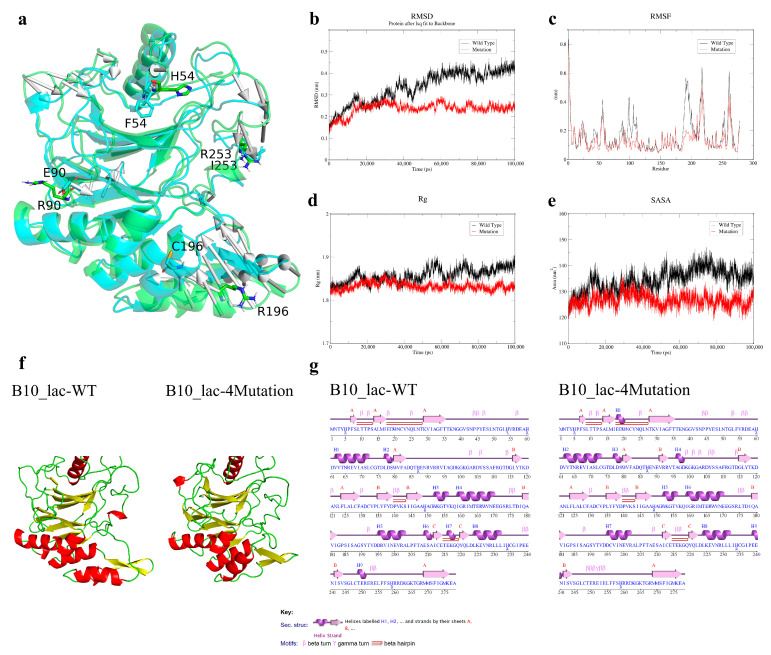
Molecular dynamics simulations and structural characterization of B10 laccase wild-type and its optimal mutant. (**a**) Spatial location and stick model of mutation sites in B10 laccase. (**b**) RMSD analysis of B10 laccase WT and the optimal mutant. (**c**) RMSF analysis of B10 laccase WT and the optimal mutant. (**d**) Rg analysis of B10 laccase WT and the optimal mutant. (**e**) SASA analysis of B10 laccase WT and the optimal mutant. (**f**) 3D structural superposition of WT and the optimal mutant. (**g**) Secondary structure analysis of B10 laccase WT and the optimal mutant.

Wild-type B10 laccase and its quadruple mutant (R90E/R196C/H54F/R253I) were subjected to 100 ns molecular dynamics simulations at 353.15 K (80 °C). Trajectory analysis showed that the wild-type protein displayed substantial RMSD fluctuations and a continued upward drift during the mid-simulation period (20–60 ns), whereas the mutant exhibited lower RMSD values and a smaller overall increase, followed by rapid convergence to a stable plateau during the mid-to-late stage of the simulation (60–100 ns) (Figure 4a,b). Comparison of residue-wise RMSF profiles showed that the quadruple mutations markedly reduced local fluctuations at the target residues (Figure 4c). In particular, the RMSF at the key mutation site ARG196 decreased from 0.4662 nm in the wild type to 0.1731 nm in the mutant, corresponding to a 62% reduction. During the 100 ns high-temperature simulation, the wild-type laccase showed a pronounced increase in Rg during the latter half of the trajectory, whereas the mutant maintained a lower Rg baseline and remained stable throughout the entire simulation (Figure 4d). For SASA, the wild type showed substantial fluctuations, reaching up to 140 nm^2^ between 60 and 80 ns, whereas the mutant remained stably within a lower range of 125–135 nm^2^ (Figure 4e). Analysis of the terminal simulation conformations using PyMOL and PDBsum showed that, at residue 90, the wild type adopted a random coil whereas the quadruple mutant evolved into an ordered β-sheet structure; at residue 253, the mutant changed from an α-helix to a random coil without inducing global destabilization; and the region surrounding residue 196 underwent marked inward compaction (Figure 4g).

### 3.5. Construction and Fermentation Optimization of a Pichia pastoris Secretory Expression System

**Figure 5:**
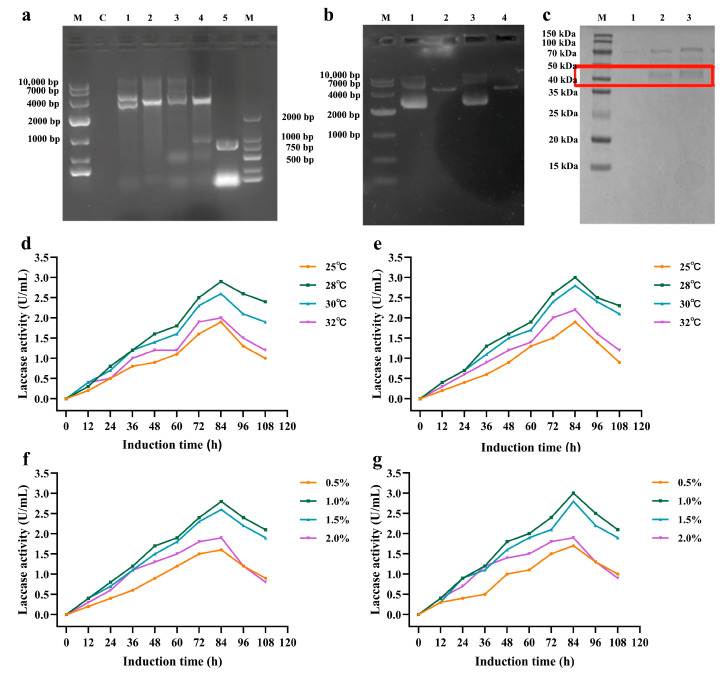
Construction of expression vectors and optimization of induction conditions for recombinant wild-type and mutant laccases in *Pichia pastoris*. (**a**) Identification of recombinant pPICZαA-WT plasmid via double digestion (M: DNA marker; 1: undigested recombinant plasmid pPICZαA-WT; 2: double-digested recombinant plasmid pPICZαA-WT; 3: undigested recombinant mutant plasmid pPICZαA-R90E/R196C/H54F/R253I; 4: double-digested recombinant mutant plasmid pPICZαA-R90E/R196C/H54F/R253I; 5: B10 laccase gene fragment; C: negative control). (**b**) Linearization of recombinant pPICZαA-WT plasmid via PmeI single digestion (M: DNA marker; 1: undigested recombinant plasmid pPICZαA-WT; 2: single-digested recombinant plasmid pPICZαA-WT; 3: undigested recombinant mutant plasmid pPICZαA-R90E/R196C/H54F/R253I; 4: single-digested recombinant mutant plasmid pPICZαA-R90E/R196C/H54F/R253I). (**c**) SDS-PAGE analysis of recombinant laccase secreted in *Pichia pastoris* culture supernatant (M: protein marker; 1: culture supernatant of wild-type Pichia pastoris GS115; 2: culture supernatant of recombinant *P. pastoris* pPICZαA-WT; 3: culture supernatant of the recombinant quadruple mutant pPICZαA-R90E/R196C/H54F/R253I; The red box highlights the protein band at approximately 35–40 kDa corresponding to the target protein). (**d**) Effect of induction temperature on the production of recombinant WT laccase in *Pichia pastoris*. (**e**) Effect of induction temperature on the production of the optimal recombinant laccase mutant in *Pichia pastoris*. (**f**) Effect of methanol concentration on the production of recombinant WT laccase in *Pichia pastoris*. (**g**) Effect of methanol concentration on the production of the optimal recombinant laccase mutant in *Pichia pastoris*.

A secretory expression platform was established using the *Pichia pastoris* GS115 strain. The gene sequences encoding wild-type B10 and the quadruple mutant were codon-optimized and cloned into the pPICZαA vector containing the AOX1 promoter and α-factor secretion signal peptide, followed by electroporation-mediated homologous recombination into the yeast genome (Figure 5a,b). Two-dimensional optimization of induction temperature (25–32 °C) and methanol concentration (0.5–2.0%) indicated that 28 °C with 1.0–1.5% methanol yielded the optimal conditions for recombinant protein secretion. After 84 h of induction, laccase activities in the culture supernatants of the wild type (pPICZαA-WT) and the quadruple mutant (pPICZαA) reached 2.9 U/mL and 3.0 U/mL, respectively (Figure 5d–g). SDS–PAGE analysis revealed a single protein band of approximately 40 kDa in the ultrafiltration-concentrated culture supernatant t (Figure 5c).

### 3.6. Verification of AFB1 Degradation by Yeast Culture Supernatant at Elevated Temperatures and in a Feed Matrix

**Figure 6:**
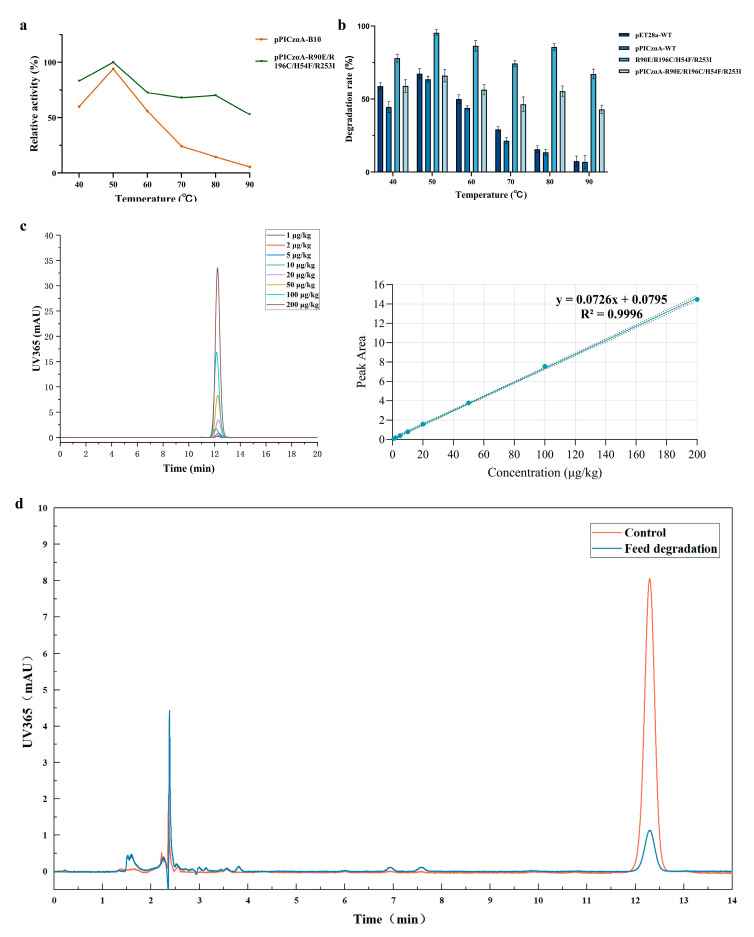
Thermostability, AFB1 degradation capacity, and practical validation of recombinant b10 laccase expressed in *Pichia pastoris*. (**a**) Thermostability of recombinant laccase expressed in *P. pastoris*. The activity of the pPICZαA-B10 at 40 °C was set to 100%, and the relative activities of the other samples were normalized accordingly. (**b**) Comparative AFB1 degradation by yeast culture supernatant and purified *E. coli* laccase. (**c**) HPLC method validation for AFB1 quantification. Representative chromatograms of AFB1 standards at 1–200 μg/kg and the corresponding calibration curve (y = 0.0726x + 0.0795, R^2^ = 0.9996). (**d**) Verification of AFB1 degradation in feed matrices at 40 °C using yeast supernatant.

The purified yeast culture supernatant was co-incubated with AFB1 (5 μg/mL) for 24 h across a temperature range of 40–90 °C. The results showed that at 80 °C the yeast-secreted wild-type laccase lost nearly all enzymatic activity, whereas the secreted quadruple mutant retained over 70% relative activity and maintained substantial AFB1 degradation capacity (Figure 6a,b). Under identical reaction conditions, the AFB1 degradation rate of the yeast-expressed mutant was slightly lower than that of the laccase purified from the *E. coli* expression system. The HPLC method used for AFB_1_ quantification in feed samples exhibited excellent linearity over the range of 1–200 μg/kg, with the calibration equation y = 0.0726x + 0.0795 and a correlation coefficient of R^2^ = 0.9996 (Figure 6c). In a separate feed-matrix experiment conducted at 40 °C, the toxin concentration in the control group without enzyme addition remained stable (approximately 50 μg/kg), indicating that the feed matrix itself did not contribute substantially to AFB1 loss under the tested conditions. By contrast, the experimental group supplemented with the yeast culture supernatant expressing pPICZαA-R90E/R196C/H54F/R253I showed a pronounced decrease in toxin concentration over time, achieving a degradation rate of up to 87.5% (Figure 6d).

## 4. Discussion

Throughout the development of enzyme engineering, overcoming the intrinsic thermostability limits of natural enzymes has fundamentally relied on reshaping their thermodynamic folding landscape. Traditional directed evolution relies heavily on approaches such as error-prone PCR and DNA shuffling. These methods require the screening of large mutant libraries, are labour-intensive and time-consuming, and are prone to becoming trapped in local fitness optima, thereby limiting substantial performance gains [20,21]. In recent years, computer-aided semi-rational design has emerged as a mainstream strategy for improving enzyme thermostability. This strategy integrates all-atom molecular dynamics simulations to identify regions of elevated flexibility under thermal perturbation, together with ΔΔG calculations for in silico saturation mutagenesis, thereby substantially reducing the unproductive search space [22,23].

In the engineering of B10 laccase, we first carried out preliminary MD simulations in aqueous solvent using the crystal structure of a highly homologous purine nucleoside phosphorylase as the template. RMSF profiles extracted from the simulation trajectories showed that, after excluding the intrinsically highly flexible disordered N-terminal region, seven prominent flexibility peaks could be identified within B10 laccase, namely LYS-217, GLU-216, ARG-196, ARG-90, ARG-253, ARG-56 and GLY-218. The mutation module was then used to calculate mutation-induced changes in folding free energy, identifying favourable substitutions with negative ΔΔG values, such as ARG196→CYS. Experimentally, the R196C single mutant retained more than 96% of its relative activity after heat treatment at 80 °C for 10 min and achieved an AFB1 degradation rate of 69.5% at 80 °C, substantially outperforming the wild type, which began to lose activity at 60 °C and was completely inactivated at 90 °C. However, the effects of single mutations in protein networks are often constrained by activity–stability trade-offs [24]. To overcome this limitation, an iterative saturation mutagenesis strategy was introduced into the engineering of B10 laccase. By using the optimal single mutant as the template for successive rounds of multi-site combination and mutation stacking, ISM enables the systematic exploitation of beneficial epistatic interactions between amino acid residues [25]. In this study, the pairwise combination of R196C with residues such as R90E identified R90E/R196C as the optimal double mutant, with a specific activity at 80 °C that was 1.25-fold higher than that of the R196C single mutant and an AFB1 degradation rate of 77.5%. A further round of MD simulation and in silico mutational analysis then identified HIS54 and ARG253 as additional beneficial sites, enabling the construction of the quadruple mutant (R90E/R196C/H54F/R253I) and establishing an effective evolutionary route towards extreme thermostability.

A comparison with recent rational and semi-rational engineering efforts targeting bacterial laccases further underscores the advantages of the ISM strategy. For instance, Liu et al. [26] engineered E186A and E186R variants of the CotA laccase from *Bacillus licheniformis* via site-directed mutagenesis. These mutants displayed broad degradation capability across 30–80 °C, with catalytic efficiencies (kcat/Km) increased by 1.8-fold and 3.2-fold relative to the wild type, and achieved AFB1 degradation rates of 82.2% and 91.8% within 12 h [26]. In another investigation targeting residue Q441 of CotA laccase, the Q441A mutation reduced steric hindrance by replacing Gln441 with the smaller Ala residue, thereby improving catalytic efficiency toward AFB1 by 1.73-fold and moderately enhancing thermostability [18]. Moreover, saturation mutagenesis of residue T418 located in the loop region surrounding the T1 copper site of *Bacillus vallismortis* frL103 laccase demonstrated that the T418A and T418S variants increased AFB1 degradation rates from 45.7% in the wild type to 56.7% and 53.6%, respectively, likely through modulation of loop flexibility and hydrogen-bond interactions [19]. Likewise, error-prone PCR combined with enrichment screening of *Bacillus pumilus* CotA laccase generated variants such as PW2 and PW4. Mutations including T262A and V426I shortened amino-acid side chains and expanded the substrate-binding cavity, whereas the G382D substitution induced conformational changes via electrostatic interactions, leading to a 2–3-fold increase in specific activity [11].

Although these studies have achieved notable improvements, most engineering efforts have focused on single- or double-site mutations located near the substrate-binding pocket, mainly aiming to reduce steric hindrance or increase local flexibility to improve catalytic efficiency. By contrast, the ISM strategy applied to B10 laccase effectively rewired the internal network of non-covalent interactions across the global conformational landscape of the protein. Previous studies have demonstrated that ISM is highly effective for improving thermostability in a variety of industrial enzymes. For instance, in the engineering of *Bacillus subtilis* lipase A, six residues with the highest B-factors in the X-ray crystal structure were identified as flexible hotspots and subjected to iterative saturation mutagenesis, yielding an optimized mutant with an approximately 20-fold longer half-life in organic solvents (Strategies for Stabilization of Enzymes in Organic Solvents). Likewise, ISM screening of flexible residues surrounding the catalytic center of *Candida antarctica* lipase B (CalB) produced the D223G/L278M variant, which exhibited a 13-fold increase in half-life at 48 °C [27]. In another example, multi-site mutation of deamidation-prone Asn residues in the alkaline protease AprE 2709 from *Bacillus licheniformis* (N61G/N160G/N211G) extended the enzyme half-life at 60 °C by 2.89-fold [28]. Moreover, in a study combining ancestral sequence reconstruction with ISM for the engineering of D-carbamoylase, the combinatorial mutant D1 displayed an increase of approximately 16 °C in the half-life temperature (T50) [29]. In this study, the quadruple mutant of B10 laccase generated through ISM effectively circumvented the activity loss often associated with rigidifying single mutations. Through cooperative effects among long-range interaction networks within the protein, the engineered enzyme simultaneously achieved exceptional conformational stability and efficient AFB1 degradation at the extreme temperature of 80 °C.

To resolve the physicochemical basis of the exceptional thermostability of the quadruple mutant (R90E/R196C/H54F/R253I) at atomic resolution, long-timescale molecular dynamics simulations under high-temperature conditions were indispensable. In 100 ns simulations at 353.15 K, wild-type B10 laccase exhibited pronounced RMSD fluctuations and a sustained increase during the middle stage of the trajectory (20–60 ns), consistent with extensive backbone distortion and the onset of thermal unfolding under strong heat stress. By contrast, both the absolute RMSD values and the magnitude of fluctuation were markedly suppressed in the quadruple mutant, whose trajectory rapidly converged and stabilized during the late stage of the simulation (60–100 ns). Moreover, the wild type displayed substantial SASA fluctuations, reaching up to 140 nm^2^ during 60–80 ns, together with a marked increase in Rg. In contrast, the mutant maintained a relatively low and stable SASA within 125–135 nm^2^, and its Rg remained lower and essentially constant throughout the simulation. Structural analysis further indicated that the region surrounding residue 90 adopted a highly flexible random-coil conformation in the wild type, whereas in the quadruple mutant it was reorganized into a more ordered β-sheet structure. In protein physicochemistry and biomaterials engineering, coil-to-β-sheet transition is widely recognized as a central mechanism underlying enhanced thermostability, mechanical resistance and protease resistance in macromolecular systems [30]. This conformational transition has been extensively documented in previous studies. For example, studies of silk fibroin materials have shown that physical shearing, thermal-field regulation or chemical crosslinking, such as glutaraldehyde treatment, can drive the transition of silk fibroin from loosely disordered random-coil conformations to highly crystalline β-sheet structures. Such conformational conversion not only increases the tensile strength of the material, but also raises its thermal degradation temperature, thereby conferring substantially improved thermal stability [31]. In self-assembling peptide systems, shielding of electrostatic interactions together with hydrophobic collapse can trigger a transition from random coil to β-sheet. The resulting three-dimensional network, reinforced by dense intra- and intermolecular backbone hydrogen bonds, can withstand severe thermal perturbation and extreme mechanical shear [32,33]. Similarly, in studies of pathological protein aggregation, including tau and amyloid proteins, the irreversible transition from random-coil conformations to β-sheet-rich states has been recognized as a major driving force for the formation of highly stable fibrillar aggregates that are resistant to cellular proteostasis mechanisms [34,35].

At the key mutation site ARG196, MD analysis showed that the RMSF value decreased sharply from 0.4662 nm in the wild type to 0.1731 nm in the mutant, corresponding to a 62% reduction. More importantly, the region surrounding residue 196 underwent a pronounced inward contraction. In naturally thermostable proteins from thermophiles, a higher core packing density than that observed in mesophilic counterparts is a hallmark feature associated with resistance to thermal denaturation [36]. At the same time, residue 253 in the mutant shifted from a relatively rigid α-helix to a random-coil conformation. This local structural relaxation provided the flexibility required for the laccase to efficiently accommodate and degrade the bulky aflatoxin substrate with substantial steric hindrance, even under extreme high-temperature conditions.

At 80 °C, most fungal laccases, including those derived from *Trametes* spp. and *Aspergillus*, are expected to undergo severe irreversible denaturation. Even among bacterial laccases, the *Bacillus vallismortis* frL103 mutant reached a mediator-free AFB1 degradation rate of only 56.7% at 37 °C [19]; free rCotA from *Bacillus subtilis* ZJ-2019-1 degraded only 41.37% of AFB1 within 12 h even at its optimal temperature of 70 °C [37]. Although recent work by Liu et al. showed that the *Bacillus licheniformis* CotA E186A/E186R mutants could also achieve mediator-free degradation rates above 90% across a broad temperature range, this engineering strategy was still primarily centered on relieving steric hindrance at a single active-site position [26]. By contrast, the B10 quadruple mutant sustained continuous degradation for 24 h at 80 °C and achieved an AFB1 degradation rate of 86.9%, despite thermal perturbation sufficiently strong to disrupt non-covalent interactions. These results strongly support the view that the previously described random-coil-to-β-sheet transition contributed substantially to the enhanced structural rigidity of the enzyme.

## 5. Conclusions

In this study, an enzyme engineering strategy integrating molecular dynamics simulation with semi-rational design successfully generated a B10 laccase variant with markedly enhanced thermostability. The optimized mutant retained strong AFB1 degradation activity at 80 °C and exhibited effective degradation performance in a simulated feed matrix. Structural analyses indicated that the enhanced thermostability was mainly associated with reduced local flexibility and a more compact global conformation. This work provides a new framework for the directed engineering of thermostable laccases and establishes a basis for detoxification-related applications of AFB1 control under high-temperature conditions.

## Data Availability

The original contributions presented in this study are included in the article. Further inquiries can be directed to the corresponding author.

## Abbreviations

The following abbreviations are used in this manuscript:

AFB1	Aflatoxin B1
ISM	Iterative Saturation Mutagenesis
MD	Molecular Dynamics
RMSD	Root Mean Square Deviation
RMSF	Root Mean Square Fluctuation
Rg	Radius of Gyration
SASA	Solvent-Accessible Surface Area
ΔΔG	Change in Folding Free Energy
WT	Wild Type
PDB	Protein Data Bank
TIP3P	Transferable Intermolecular Potential with 3 Points
NVT	Constant Number of particles, Volume, and Temperature ensemble
NPT	Constant Number of particles, Pressure, and Temperature ensemble
PMSF	Phenylmethylsulfonyl Fluoride
ABTS	2,2′-Azino-bis(3-ethylbenzothiazoline-6-sulfonic acid)
HPLC	High-Performance Liquid Chromatography
AOX1	Alcohol Oxidase 1
